# Experimental techniques for the characterization of carbon nanoparticles – a brief overview

**DOI:** 10.3762/bjnano.5.186

**Published:** 2014-10-13

**Authors:** Wojciech Kempiński, Szymon Łoś, Mateusz Kempiński, Damian Markowski

**Affiliations:** 1 Institute of Molecular Physics, Polish Academy of Sciences, Mariana Smoluchowskiego 17, Poznań, 60-179, Poland; 2 Faculty of Physics, Adam Mickiewicz University, Umultowska 85, Poznań, 61-614, Poland; 3 NanoBioMedicalCentre, Adam Mickiewicz University, Umultowska 85, Poznań, 61-614, Poland

**Keywords:** carbon nanoparticles, charge carrier transport, host–guest interactions, spin localization

## Abstract

The review of four experimental methods: X-ray diffraction, Raman spectroscopy, electron paramagnetic resonance and four-point electrical conductivity measurements is presented to characterize carbon nanoparticles. Two types of carbon nanoparticle systems are discussed: one comprising the powder of individual carbon nanoparticles and the second as a structurally interconnected nanoparticle matrix in the form of a fiber. X-ray diffraction and Raman spectroscopy reveal the atomic structure of the carbon nanoparticles and allow for observation of the changes in the quasi-graphitic ordering induced by ultrasonic irradiation and with the so-called quasi-high pressure effect under adsorption conditions. Structural changes have strong influence on the electronic properties, especially the localization of charge carriers within the nanoparticles, which can be observed with the EPR technique. This in turn can be well-correlated with the four-point electrical conductivity measurements which directly show the character of the charge carrier transport within the examined structures.

## Review

### Introduction

Quasi-graphitic carbon nanoparticles (CNs) were found to show very interesting behavior with respect to the localization of charge carriers. Due to their large specific surface area, CNs are very sensitive to the adsorption of various molecules. Their electronic properties strongly depend on their structure and interactions between the molecules adsorbed in pores and the pore walls (host–guest interactions) and this behavior is the main interest of this work. It was shown that it is possible to control the charge carrier transport within the systems of CNs by varying the temperature, adsorbed molecules and external electric field. Due to the significant changes in resistivity induced by the host–guest interactions, the systems of CNs might prove interesting in the fields of gas sensing, molecular electronics or spintronics. Localization of charge carriers (spins) within the CNs is the crucial phenomenon for such applications [1–3]. Dresselhaus’ group described the process of charge carrier transport within the carbon nano-textures using two models – Coulomb gap variable range hopping (CGVRH) and charge energy limited tunneling conduction (CELTC) [4–6]. The latter model was also used by other researchers to describe disordered carbons [7]. Both models originate from the well-explored system of granular metals in which potential barriers are created with different dielectric separators [8]. The possibility to control the charge carrier transport (“tunable electrical conductivity”) was shown in porous metal-organic frameworks (MOFs) with adsorbed guest molecules [9]. It was also shown that by appropriate choice of guest molecules, it is possible to control the charge (spin) transport in the nano-graphitic or graphene-like systems [10–11].

In this review we have collected results from four experimental methods: X-ray diffraction (XRD), Raman spectroscopy, electron paramagnetic resonance (EPR) and four-point electrical conductivity measurements, in order to characterize the two CN systems. These well-defined nanoparticle powders were comprised of separated CNs and a fiber formed by randomly connected CNs, referred to as the activated carbon fiber herein. With a comprehensive study of such systems, comprising the research of host–guest interactions of CNs with different molecules, the problem of controlled charge and spin localization within different systems based on nano-graphite or nano-graphene crystallites can be approached.

### Individual carbon nanoparticles – the structure

**Figure 1:**
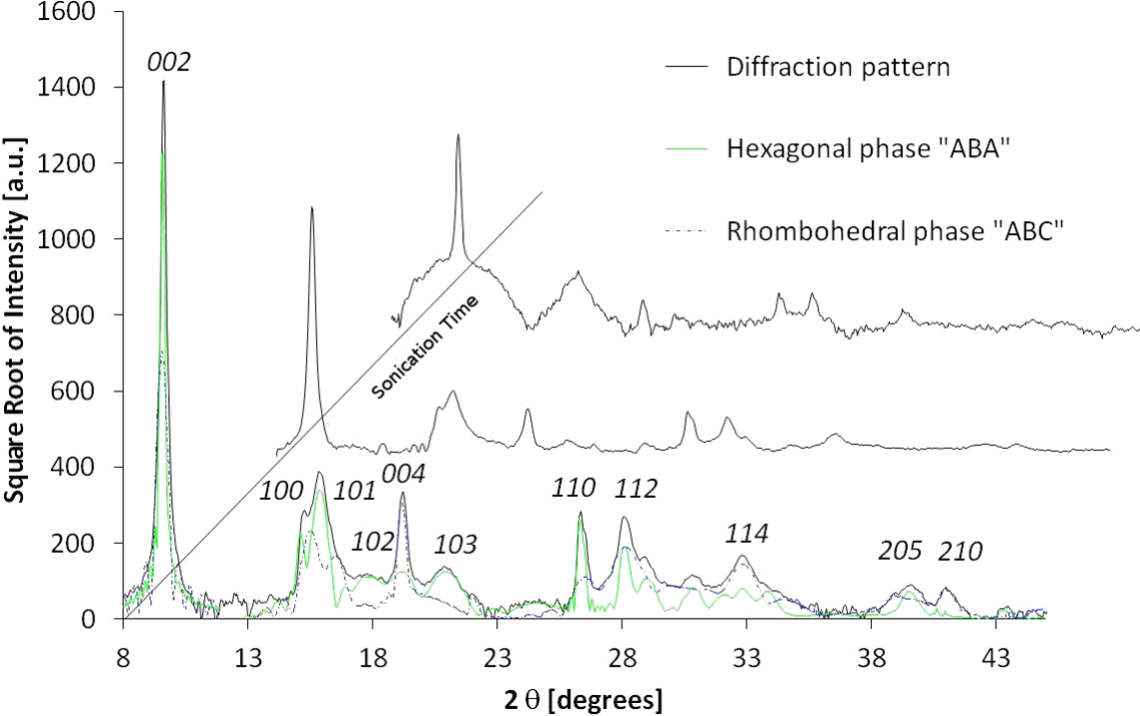
The experimental XRD diffraction pattern obtained for ultrasound-treated graphite. Both hexagonal and rhombohedral phase have been assumed to simulate the diffraction pattern. For clarity, the Miller indices have been assigned only to the hexagonal graphite phase.

Graphite crystals are layered materials with the strong carbon bonding within the atomic sheets and weak interaction between them [12]. It is relatively easily to pulverize this material to form CNs. XRD and Raman spectroscopy are suitable methods for crystal size approximation [13–15]. To obtain CNs similar to those forming activated carbon fibers described in the next paragraph, crystals of hexagonal graphite were treated with ultrasonic irradiation [16]. This procedure results in the development of an internal strain which generates the stacking fault by shifting the layers laterally as well as increasing the distance between them. The in-plane coherence length and the degree of three dimensional order of the crystallites can be calculated from the powder XRD data. The diffractograms presented in Figure 1 show a progressive decrease in the peak intensity as well as broadening of the linewidth. The most prominent peak of the hexagonal structure, (002), undergoes the strongest alteration. The intensity proportional to the number of atomic layers is gradually reduced with the irradiation time. Additionally, broadening of the peak width is observed due to the reduction of the coherence length. This effect is observed for all peaks present. This is a signature of the decay of the periodicity in the crystal, especially along the crystallographic *c* direction perpendicular to the graphene layers. Translation of these layers is the mechanical consequence of ultrasonic radiation. An accurate analysis reveals the presence of a set of extra peaks whose positions suggest the occurrence of a rhombohedral phase [17]. It was shown that this phase can be generated locally by grinding the graphite [18–20] and constitutes up to 40% volume content of the sample. Figure 1 presents the diffractograms of pre-grinded graphite, irradiated with ultrasonic waves. The XRD pattern simulation (peak intensity and width) gives the average crystallite thickness calculated for the major reciprocal in-plane directions. The average domain dimension, *L_a_*, can be calculated from the [100]* and [110]* directions, and coherent domain size, *L_c_* along the *c* axis, from the [002]* direction. These values obtained from Fraunhofer analysis could also be calculated from the linewidth by Scherrer’s equation [21]. Notwithstanding, in samples which contain the hexagonal and rhombohedral phases, the peaks which are characteristics of both phases overlap, resulting in a broadened linewidth, which may result in the over-estimation of the aforementioned values when compared to the samples comprising only one phase. The sonication procedure results in multiple mechanical effects such as: the disintegration of the three dimensional order, the separation of layers, the turbostratic structure formation, and the reduction of the layer size due to breaking of the C–C aromatic bonds. The presence of the two-dimensional graphene particles could not be observed with XRD.

**Figure 2:**
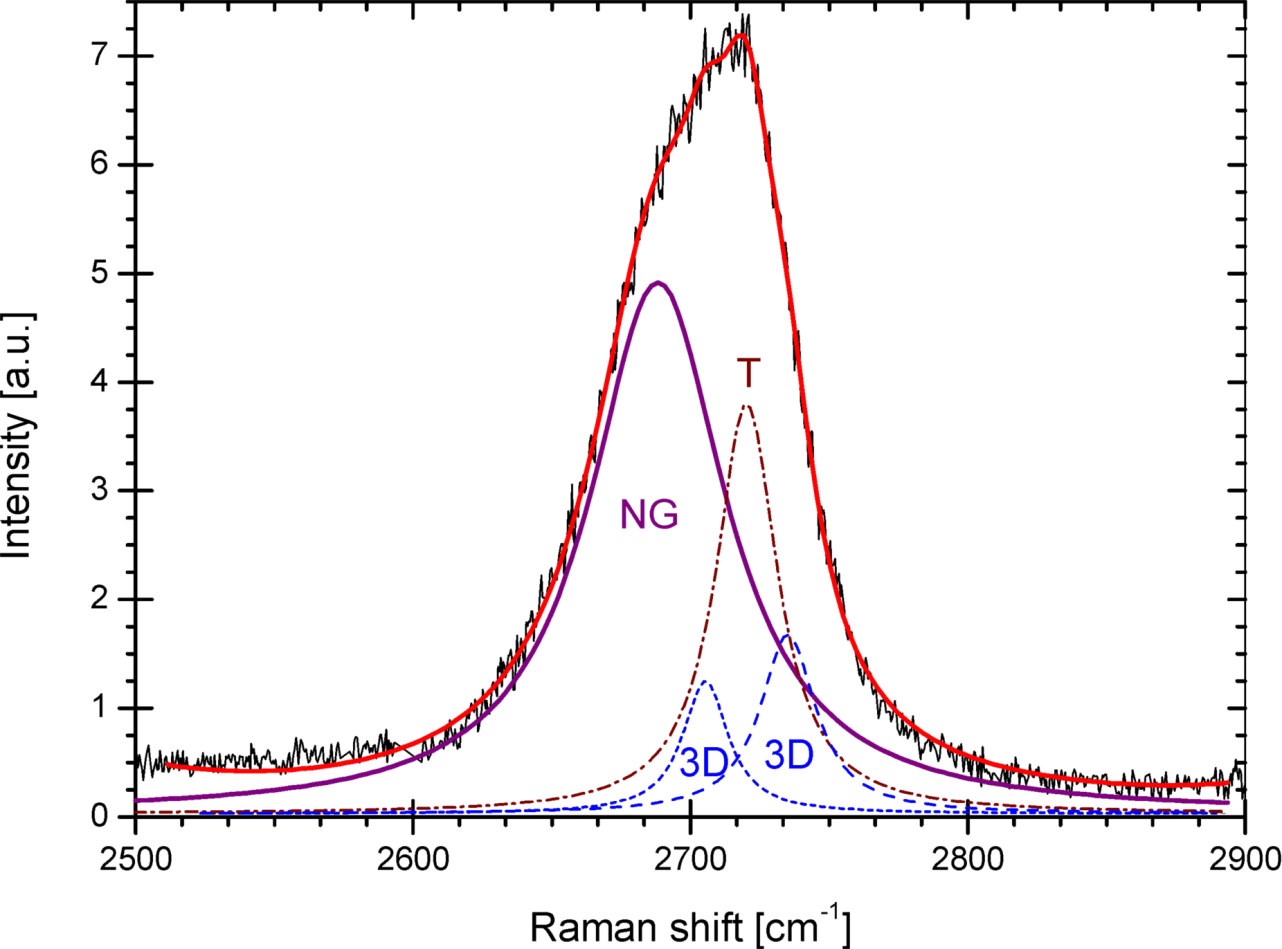
A typical second-order Raman spectra of an irradiated sample: the turbostratic (T) structure band (dotted line), the two peaks of the three dimensionally ordered graphite (3D) structure (dashed line), and the nano-graphite signal (solid line).

First- and second-order Raman spectroscopy is a useful method which allows characterization of the powders from the point of view of CNs. This technique is based on the phonon scattering phenomena and much valuable information can be obtained from the relative intensities of the spectral components. It was shown by Tuinstra and Koenig in 1970 [22–23] and recently by Ferrari et al. [14,24] and Cançado et al. [25] that the D to G peak intensity ratio in the first order spectra can be correlated with the in-plane size of the crystallites. The D peak intensity depends on the defect concentration and indicates the presence of a crystalline border [26]. It is suppressed by the perfectly organized carbon layer since it is a hexagonal ring breathing mode [14]. Second order Raman spectra are presented in Figure 2. They are composed of the three different signals because the laser irradiates sample regions with different degrees of structural ordering. The first signal represents the well-organized graphite structure (3D structure) and is comprised of two peaks in the vicinity of 2697 cm^−1^ and 2728 cm^−1^. This signal reconstruction was performed with the 1:2 amplitude ratio, respectively [27]. The second signal (marked with T) comes from the turbostratic fraction and is represented by the single Lorentzian line at 2710 cm^−1^ [28]. Each band from the above mentioned carbon types has a full width at half-maximum of approximately 30 cm^−1^. For the sonicated sample, a third signal appears (marked with NG). Its position is shifted towards shorter wavenumbers. A similar peak was previously observed on graphene treated with an argon ion beam [29]. The width of the NG line is twice as broad as the width of the 3D and T signals. This can be due to the large number of defects. This result points at the creation of a large number of defects due to the ultrasonic treatment. The NG signal can also be interpreted as the line associated with few-layered graphene flakes since the signal of double-layered graphene flakes consists of four Lorentzian lines [30] and resembles a single broad line [31]. Furthermore, triple-layered graphene would result in six peaks, and so forth. Considering this, the broad peak at 2679 cm^−1^ might be inhomogeneously broadened and can be attributed to the strained nano-graphite appearing in the sample.

There are also other ways to determine the dimensions of the CNs. Among them are impedance measurements [32] and the EPR technique performed on the conducting electrons [33]. A more detailed analysis is provided in the next section.

### Carbon nanoparticle matrix

The electric conductivity measurements were performed for activated carbon fibers (ACFs) formed from the quasi-graphitic CNs, which are mechanically connected by carbon chains or graphene fragments [36]. It was shown that the conductivity of such a system depends on the thermal energy allowing the hopping of charge carriers between the CNs [4,37]. Hopping charge carrier transport in the granular structures is described with the CGVRH model [4], from which the following equation has been derived:

[1]
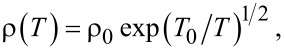


where ρ stands for the resistivity, ρ_0_ is a temperature-independent constant, *T* is the temperature and *T*_0_ is the energy needed for hopping of charge carriers.

In the low temperature regime (below 200 K) the amount of thermal excitations drastically decreases and charge carriers cannot overcome the potential barriers between the nano-crystallites and thus become localized within them [8]. The localization of charge carriers can be easily observed with the EPR technique [36]. This consists of the resonant absorption of microwave energy by unpaired spins (localized charge carriers) placed in the external magnetic field.

**Figure 3:**
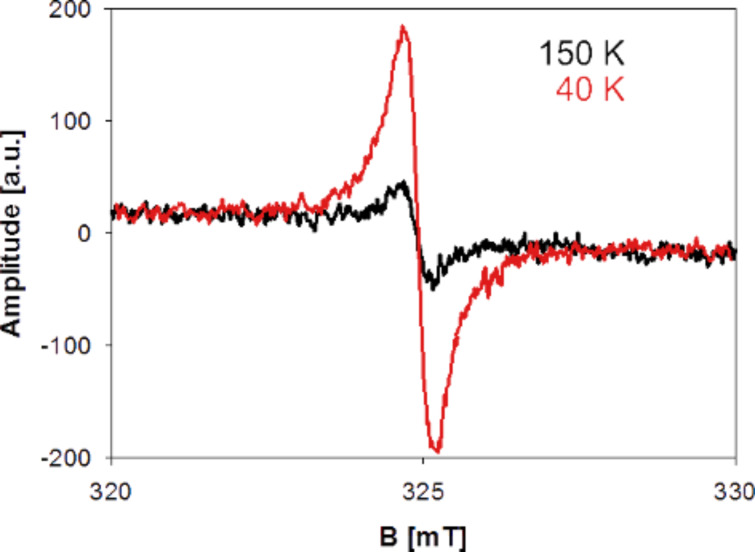
EPR signal of ACFs in different temperatures.

The EPR signal of ACFs can be observed only in the low temperature regime and its integral intensity significantly increases with the reduction of temperature [36], as represented in Figure 3.

It was shown that this increase results from the Curie’s law behavior, where integral intensity is inversely proportional to temperature, which is normally observed for paramagnetic systems with the fixed number of spins (Langevin paramagnetism) [36]. In the case of the ACFs, the number of spins observed in EPR changes according to Equation 1. This is related to the CGVRH model, where resistivity depends on the temperature. In the classical approach [37] the resistivity ρ (which is the reverse of conductivity σ) depends on the number of charge carriers *N*, their charge *e*, and mobility μ according to the equation:

[2]
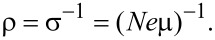


**Figure 4:**
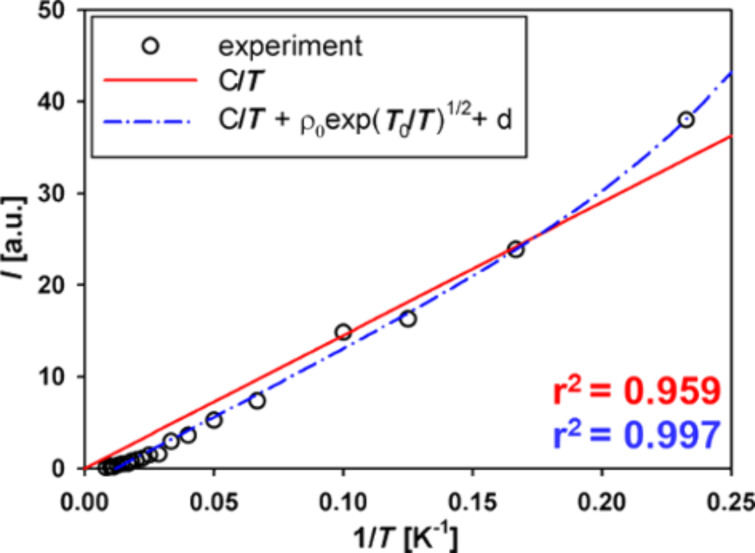
EPR signal intensity vs temperature fitted with the unmodified Curie’s law (red) and Curie’s law modified with Equation 1 (blue).

*N* decreases because charge carriers are localized within the system, thus the resistivity increases. The carriers localized within the individual CNs no longer take part in the charge transport and add to the EPR signal instead. Thus, Curie’s law must be modified with the component resulting from the Equation 1 to take into account the changing number of localized spins. This result is presented in Figure 4 [36].

**Figure 5:**
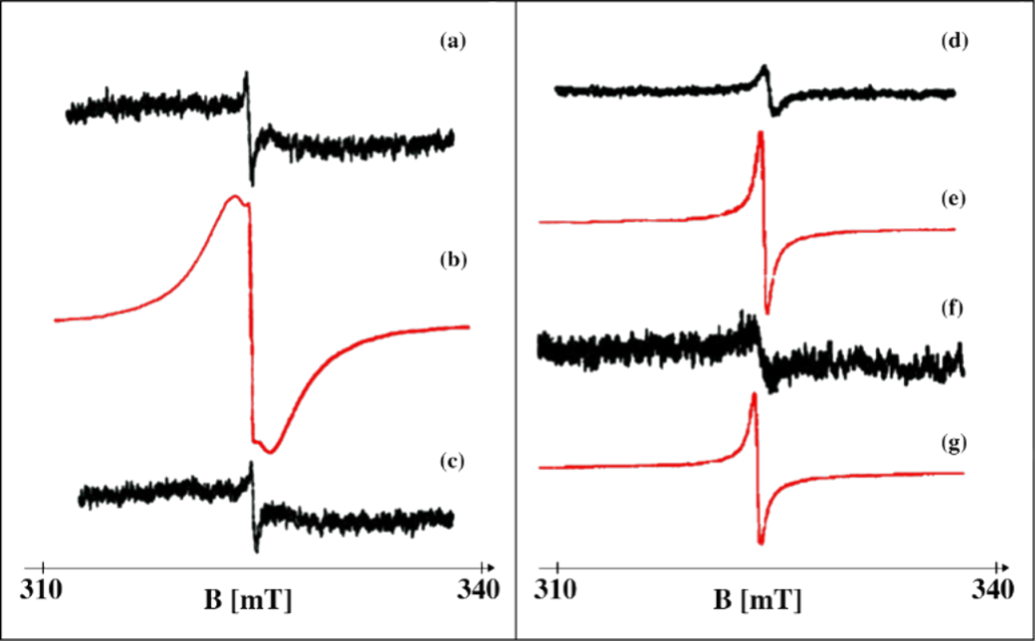
EPR signal of ACFs. Adsorption/desorption of H_2_O: (a) initial signal of pure ACFs, signal gain 4∙10^4^; (b) ACFs + H_2_O, s. g. 2∙10^3^; (c) ACFs after heating in vacuum (100 °C, 10^-4^mbar), s. g. 4∙10^4^. Adsorption/desorption of CCl_4_ (d) initial signal of pure ACFs, s. g. 2∙10^4^; (e) ACFs + CCl_4_, s. g. 2.5∙10^3^; (f) ACFs after CCl_4_ desorption, s. g. 1∙10^5^; (g) ACFs + CCl_4_, s. g. 2∙10^3^.

Another factor greatly influencing the EPR signal of ACFs is the adsorption of molecules at the surface of CNs which comprise the pore walls. The adsorption in ACF pores is of a physical nature (van der Waals forces), without any covalent bond formation. Fully reversible physisorption has been observed with EPR (see Figure 5).

The EPR spectrum of ACFs becomes strongly modified after the adsorption of guest molecules. Pure ACFs show a single narrow Lorentzian line, while the filled ones show the same narrow line plus two additional broad components in the EPR spectrum [35,38].

It was concluded that the three different lines originate from the different parts of the fibers, that is, areas not accessed by guest molecules, the pore walls and the fiber surface [35,38].

Figure 5 shows that the influence of various adsorbed molecules is varied. For example, water causes a more significant change of the EPR spectrum of the ACFs than CCl_4_. This effect is particularly visible in the experiment where the number of spins localized due to the interaction of CNs with H_2_O and CCl_4_ molecules is estimated. In this case, water causes six-times stronger localization compared to CCl_4_ [39].

The influence of H_2_O and CCl_4_ on the ACF properties is compared in several other experiments and theoretical approaches, as presented below [40–45].

XRD measurements show that guest molecules cause a strong change in the distance between graphene layers comprising the CNs in the ACF texture. Here, the lattice constant decreases from 3.78 Å (recorded for pure ACFs) to 3.33 Å and 3.30 Å for CCl_4_- and water-filled fibers, respectively. Such significant changes appear due to the so-called quasi-high pressure effect, which appear in confined liquids (e.g., adsorbed inside small pores). This causes swelling of pores of a certain width due to the decrease of the CN size [40–41]. Water seems to cause a slightly larger shrinkage of the CNs than CCl_4_, probably due to the large difference in wetting of graphitic pore walls by both liquids. The wetting in porous materials is commonly described with the microscopic wetting parameter α [42], which shows the interplay between the interactions within the liquid and of the liquid within the pore walls. The wetting parameter is given as:

[3]
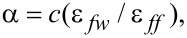


where *c* is a constant that comprises the parameters related to the structure of the pore walls and ε is the energy parameter in the Lennard–Jones potential, where the index *fw* represents the fluid–wall, and *ff* the fluid–fluid interaction. For α ≥ 1.15, the system is considered wet, while for α ≤ 1.15 it is considered non-wet. The α value for CCl_4_ in graphite is 1.9 while for water in graphite it is 0.48. A low α results in the strong hydrophobicity of the CN surface (ACF pore walls) [42].

Similar effects were observed in the spin susceptibility χ measurements, where adsorption of molecules containing the –OH groups (water amongst them) caused a larger increase in the χ-value than the adsorption of other molecules (such as CCl_4_) [43].

A more significant difference between ACFs + H_2_O and ACFs + CCl_4_ was observed in the conductivity measurements, which clearly show that the dipolar molecules cause greater increase of the *T*_0_ value than those without a dipole moment [44]. This concept is presented below.

**Figure 6:**
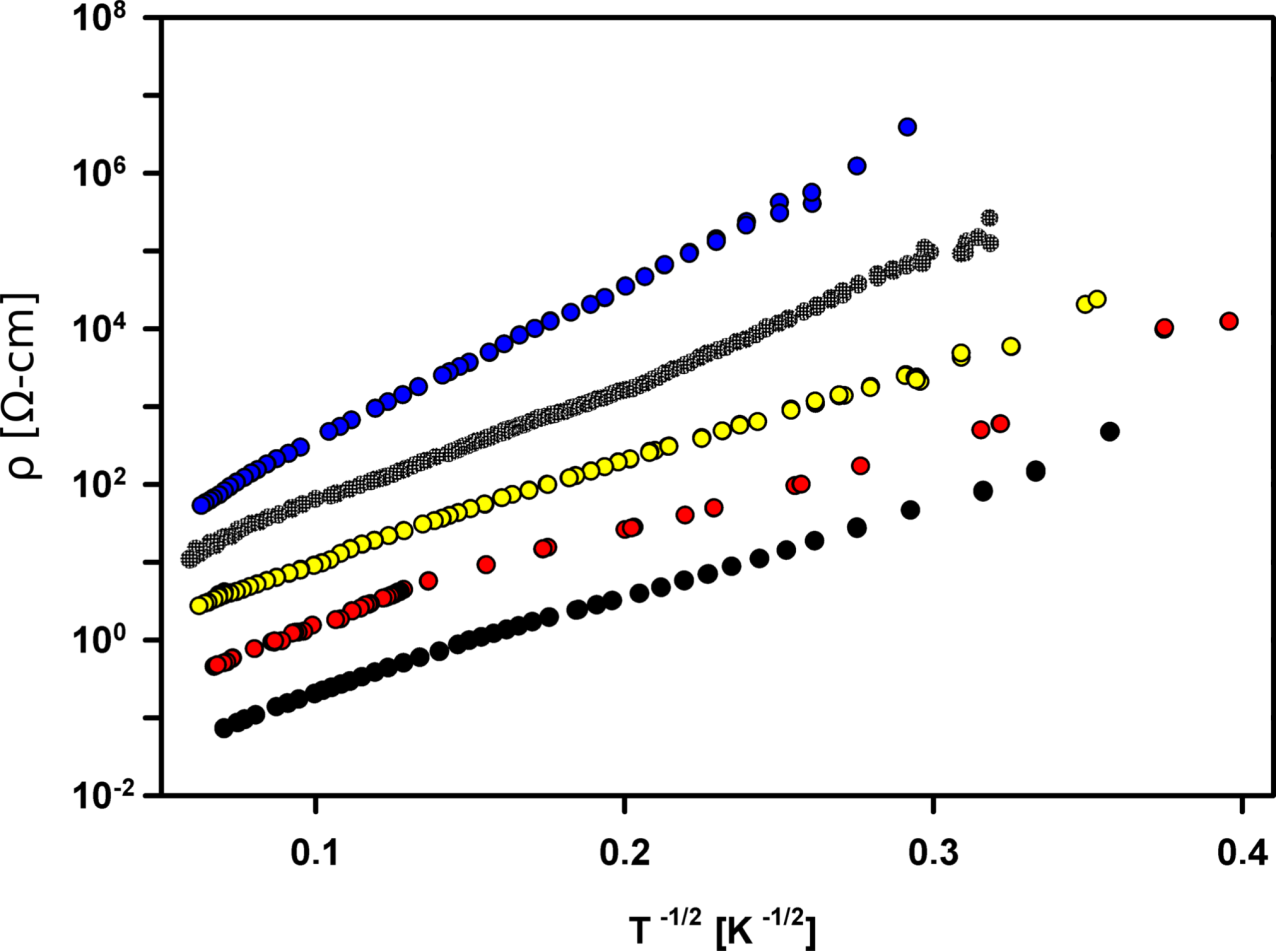
Resistivity vs the reciprocal square root of temperature for pure ACFs and ACFs filled with dipolar molecules (H_2_O, D_2_O and C_6_H_5_NO_2_). This was adapted from a previous publication [38] with the addition of the result for ACFs + non-dipolar molecules (CCl_4_). Black: pure ACF with *T*_0_ = 809 K; red: ACF + CCl_4_ with *T*_0_ = 844 K; yellow: ACF + H_2_O with *T*_0_ = 876 K; green: ACF + D_2_O with *T*_0_ = 1282 K and blue: ACF + C_6_H_5_NO_2_ with *T*_0_ = 2162 K.

It was proposed to consider the system of mechanically connected CNs as a quantum dots matrix [35,44]. In such a matrix, where CNs are structurally connected by some mechanical linkages (carbon chains or graphene fragments [34]), there exists a system of potential barriers, similar to the those formed by a dielectric separator in granular metals. The barriers depend on the dielectric constant of the separator [4,8] and in ACFs its value seems to be influenced by the existence of guest molecules inside the pores. It has been shown that the *T*_0_ parameter increases with the value of the dipole moment of the guest molecules adsorbed within the ACF pores [44]. This behavior is consistent with the CELTC model. Recent measurements of ACFs + CCl_4_ confirm this idea and Figure 6 shows that there is a clear difference between the influence of dipolar and non-dipolar guest molecules, represented by the value of the *T*_0_ parameter.

Another interesting feature is the “switching effect”, which was reported for CN texture of ACFs [36,45]. An external electric field causes the sudden increase of the resistivity of ACFs filled with dipolar guest molecules. It confirms that the value of the *T*_0_ parameter is influenced by the local electric fields created in the vicinity of individual CNs.

## Conclusion

According to the above considerations it can be concluded that different systems of interconnected quasi-graphitic (or graphene-like) CNs may form a good basis for detectors of molecules and atoms of different types, as their electronic properties are very sensitive to adsorption. Another very interesting feature of the CN textures is the possibility to control certain physical properties of the carbon nanoparticle systems by choosing the specific molecules. The ability to control the structure and the charge or spin transport in nanostructured materials can be very useful from the point of view of many future applications.
